# Chromosome-level Genome of the Muskrat (*Ondatra zibethicus*)

**DOI:** 10.1093/gbe/evac138

**Published:** 2022-09-16

**Authors:** Haimeng Li, Minhui Shi, Qing Wang, Tian Xia, Sunil Kumar Sahu, Yu Zhang, Jiangang Wang, Tianfeng Li, Yue Ma, Tianlu Liu, Huan Liu, Tianming Lan, Suying Bai

**Affiliations:** College of Life Sciences, University of Chinese Academy of Sciences, Beijing 100049, China; BGI Life Science Joint Research Center, Northeast Forestry University, Harbin, China; State Key Laboratory of Agricultural Genomics, BGI-Shenzhen, Shenzhen 518083, China; College of Life Sciences, University of Chinese Academy of Sciences, Beijing 100049, China; State Key Laboratory of Agricultural Genomics, BGI-Shenzhen, Shenzhen 518083, China; College of Life Sciences, University of Chinese Academy of Sciences, Beijing 100049, China; State Key Laboratory of Agricultural Genomics, BGI-Shenzhen, Shenzhen 518083, China; The Ninth Institute of Intelligence Research, YunJi Intelligent Engineering Co., Ltd., Shenzhen, China; State Key Laboratory of Agricultural Genomics, BGI-Shenzhen, Shenzhen 518083, China; College of Wildlife and Protected Area, Northeast Forestry University, Harbin 150040, China; State Key Laboratory of Agricultural Genomics, BGI-Shenzhen, Shenzhen 518083, China; College of Wildlife and Protected Area, Northeast Forestry University, Harbin 150040, China; BGI Life Science Joint Research Center, Northeast Forestry University, Harbin, China; College of Wildlife and Protected Area, Northeast Forestry University, Harbin 150040, China; BGI Life Science Joint Research Center, Northeast Forestry University, Harbin, China; State Key Laboratory of Agricultural Genomics, BGI-Shenzhen, Shenzhen 518083, China; Guangdong Provincial Key Laboratory of Genome Read and Write, BGI-Shenzhen, Shenzhen 518120, China; BGI Life Science Joint Research Center, Northeast Forestry University, Harbin, China; State Key Laboratory of Agricultural Genomics, BGI-Shenzhen, Shenzhen 518083, China; College of Wildlife and Protected Area, Northeast Forestry University, Harbin 150040, China

**Keywords:** chromosome-level genome, muskrat, sex chromosome, musk

## Abstract

The muskrat (*Ondatra zibethicus*) is a semi-aquatic rodent species with ecological, economic, and medicinal importance. Here, we present an improved genome assembly, which is the first high-quality chromosome-level genome of the muskrat with high completeness and contiguity assembled using single-tube long fragment read, BGISEQ, and Hi-C sequencing technologies. The genome size of the final assembly was 2.63 Gb with 27 pseudochromosomes. The length of scaffold N50 reached 80.25 Mb with a Benchmarking Universal Single-Copy Ortholog score of 91.3%. We identified a 66.98 Mb X chromosome and a 1.14-Mb Y-linked genome region, and these sex-linked regions were validated by resequencing 32 extra male individuals. We predicted 19,396 protein-coding genes, among which 19,395 (99.99%) were functionally annotated. The expanded gene families in the muskrat genome were found to be enriched in several organic synthesis- and metabolism-related Gene Ontology terms, suggesting the likely genomic basis for the production and secretion of musk. This chromosome-level genome represents a valuable resource for improving our understanding of muskrat ecology and musk secretion.

**Significance**

This is the first high-quality chromosome-level muskrat genome with high genome contiguity, completeness, and genome annotation, and it provides a useful genomic resource for genome-wide screening related to the genomic basis of musk production and secretion as well as the ecological management of the muskrat as an invasive species.

## Introduction

The muskrat (*Ondatra zibethicus*; Rodentia: Cricetidae) is a medium-sized rodent that is the only species in *Ondatra.* It is also known as the water rat because it has adapted to live semi-aquatic lifestyle, inhabiting wetlands, ponds, coastal areas, lakes, river banks, and estuaries (Schuster et al. 2021). The muskrat is a highly adaptable species that is native to North America and Canada but has been introduced to Europe, Asia, South America, and Australia (Gintarė Skyrienė and Paulauskas 2012). In China, muskrats were first found in the Heilongjiang border region in the 1950s after they were introduced from the former Soviet Union (Zhang et al. 2020). Although *O. zibethicus* is considered an invasive species in Europe and Asia, including France, Germany, Poland, Russian, Mongolia, etc. and is thought to be harmful to local ecosystems (Gintarė Skyrienė and Paulauskas 2012), it is also known to have positive effects that help protect ecosystems. In wetland ecosystems, the muskrat is an influential herbivore that strongly affects aquatic vegetation, whereas muskrats are also prey for several carnivores (Ward et al. 2021). Therefore, *O. zibethicus* is an important ecohydrological indicator species (Ward et al. 2021), and increases and decreases in its population are closely related to the changes in floodplains (Ward et al. 2019). Despite the ecological significance of the species, the genomic background of *O. zibethicus* is poorly characterized; thus, obtaining the *O. zibethicus* genome will be important for elucidating the genetic mechanisms underlying the species’ distinct biological characteristics.

The muskrat has economic and medicinal value related to its meat and fur but especially its musk (Liu et al. 2019). Male muskrats possess a pair of specialized scent glands between the skin and muscles near their tail that produce a yellowish substance similar to the musk secreted by musk deer (van Dorp et al. 1973; Li et al. 2017). Indeed, the common name of the muskrat is derived from its musk secretion (Cao et al. 2015). The components of muskrat musk are reportedly similar to those of musk deer musk, with the key components including l-muscone and some macrocyclic compounds, such as civetone, cyclododecanone, cyclopentadecanone, fatty acids, esters, and sterol compounds (van Dorp et al. 1973; Li and Song 1994; Lee et al. 2019). Musk deer musk is an essential component of Woohwangcheongsimwon, which is used to prevent and treat stroke, palpitations, hypertension, unconsciousness, and convulsions (Kim et al. 2008). However, the trade of musk deer musk is now prohibited according to the Convention on International Trade in Endangered Species of Wild Fauna and Flora (Lee et al. 2019). Muskrat musk is an ideal substitute for musk deer musk and would be easily obtained because muskrats are easy to manage and breed. Musk of muskrat can be used to treat stroke, swelling, and abscesses because it relieves pain, reduces inflammation, and activates blood (Lee et al. 2019). The scent gland of the muskrat exhibits seasonal changes that are closely related to its reproduction. From March to October, the volume of the scent gland increases substantially and a large amount of musk is secreted. However, the scent gland starts to atrophy and is replaced by adipose tissue from October; consequently, musk is not secreted from October to March the next year (Li et al. 2017). However, the genomic basis for the seasonal changes in the muskrat scent gland is not yet clarified.

Here, we assembled the first chromosome-scale genome of the muskrat using single-tube long fragment read (stLFR; Wang et al. 2019) and Hi-C (Belton et al. 2012) technologies. Our assembly shows improved contiguity compared with that of a genome published previously (Zhou et al. 2020). In particular, we identified sex-linked genome regions, which may be closely related to the seasonal changes in muskrat reproductive activities. This improved chromosome-scale genome represents a valuable resource for improving our understanding of muskrat ecology and musk secretion.

## Results and Discussion

### Chromosome-level Genome Assembly

**Fig. 1.**
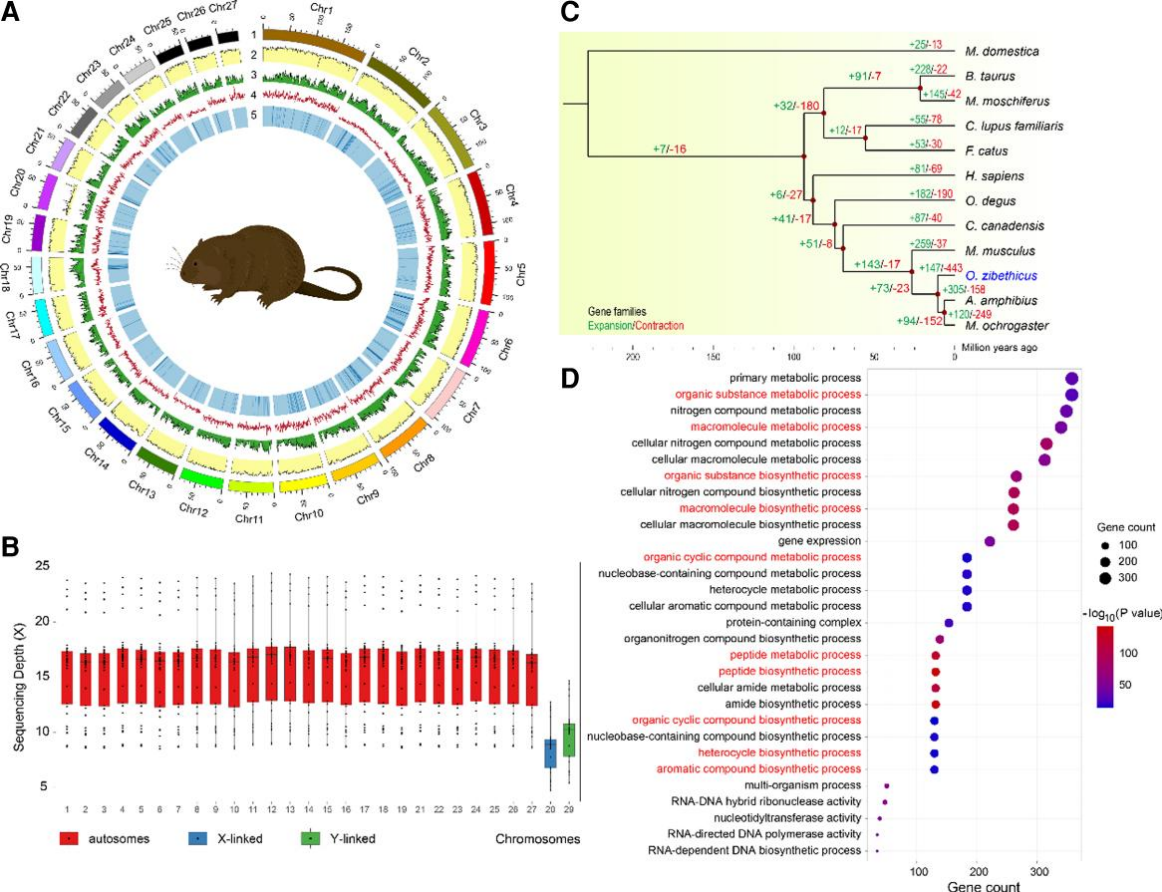
Genome landscape of the muskrat genome, comparative genomics analysis, and enrichment analysis of expanded gene families. (*A*) Overview of the chromosome-scale genome of the muskrat. (1) The 27 chromosomes; (2) read depth mapped to the genome; (3) GC content; (4) repeat density; and (5) gene density. (*B*) Identification of Ylinked regions and the X pseudochromosome. The sequencing depth of the sex-linked genome regions is nearly half that of the autosomes. (*C*) Divergence time estimation and the inference of expanded/contracted gene families. Green and red numbers on each node represent the number of expanded and contracted gene families, respectively. (*D*) Significantly enriched GO terms in the muskrat genome.

The estimated genome size of *O. zibethicus* was 2.69 Gb based on the frequency of 21-mer using short BGISEQ reads (supplementary fig. S1, Supplementary Material online). First, we generated a 2.71-Gb genome with a scaffold N50 of 5.07 Mb using 212.90 Gb of stLFR sequencing data (table 1). Subsequently, 542.59 Gb of Hi-C sequencing data was used for concatenating the primary scaffolds in a chromosome-level assembly. The final genome assembly was 2.63 Gb with 2.33 Gb genome regions assigned to 27 pseudochromosomes, which is consistent with a karyotypic study (2*n* = 54 (table 1, supplementary table S1 and fig. S2, Supplementary Material online; Pizzimenti 1971). We also identified a 66.98-Mb X chromosome and 1.14 Mb Y-linked regions in this genome by screening sex-linked genes across scaffolds (fig. 1*A*). Both the X chromosome and Y-linked regions were validated using 32 additional male individuals with lower sequencing depth than that of the autosomes (fig. 1*B*). The identification of sex-linked genomic regions provides a basic resource for exploring musk secretion in male muskrats. In total, 91.3% of 9,926 mammalian genes were complete in the muskrat genome according to Benchmarking Universal Single-Copy Orthologs (BUSCO) analysis (supplementary table S2, Supplementary Material online). In addition, 99.66% and 98.97% of reads and BGISEQ short reads and Hi-C reads, respectively, could be mapped to the genome assembly in this study. Taken together, these findings indicate that our improved genome assembly of muskrat is high quality, contiguous, and complete at the chromosome level.

**Table 1:** Genome Assembly and Annotation Data Related to the Muskrat Genome Assembled in This Study

Item	Category	Number
Sequencing data	stLFR (Gb)	212.90
	WGS (Gb)	130.28
	Hi-C (Gb)	542.59
	Resequencing (32 individuals) (Gb)	1379.49
	RNA-seq (Gb)	105.29
Assembly (stLFR)	Estimated genome size (Gb)	2.69
	Assembled genome size (Gb)	2.71
	Karyotype	2*n* = 54
	Contig N50 (Kb)	56.15
	Longest scaffold (Mb)	34.52
Assembly (Hi-C)	Assembled genome size (Gb)	2.63
	Scaffold N50 (Mb)	80.25
	Longest scaffold (Mb)	196.46
Annotation	GC content (%)	37.8
	Repeat sequences (%)	34.32
	Number of protein-coding genes	19,396
	Number of functional annotated genes	19,395
	Average gene length (Kb)	31.92
	Average exon length (bp)	181.71
	Average intron length (Kb)	3.90
	Average exon per gene	8.78

### Genome Annotation

In total, 904.51 Mb repetitive elements were identified in our assembled *O. zibethicus* genome, accounting for 34.37% of the final genome size (supplementary table S3, Supplementary Material online). Long interspersed nuclear elements were the most dominant repeat element (450.22 Mb), followed by LTRs (169.68 Mb), DNA (3.88 Mb), and short interspersed nuclear elements (126.24 Mb; supplementary tables S4 and S5, Supplementary Material online). All repetitive elements were masked for gene annotation. We predicted 19,396 protein-coding genes with high confidence by combining evidence from homology-based prediction, transcriptome alignment, and ab initio prediction (supplementary table S6, Supplementary Material online). The average gene length, exon length, and intron length were 31.92 kb, 181.71 bp, and 3.89 kb, respectively, which is consistent with other closely related animals (table 1, supplementary fig. S3, Supplementary Material online). BUSCO analysis showed that 90.0% and 1.3% of complete and fragmented BUSCO genes were identified, respectively, indicative of a high-quality gene set (supplementary table S2, Supplementary Material online). In at least one of the five databases used in this study (supplementary fig. S4, Supplementary Material online), 19,395 (99.99%) genes were functionally annotated (table 1, supplementary table S6, Supplementary Material online). Additionally, 775 miRNAs, 3685 tRNAs, 618 rRNAs, and 1559 snRNAs were predicted in the *O. zibethicus* genome (supplementary table S7, Supplementary Material online).

### Phylogenetic Analysis and Gene Family Evolution

We performed a comparative genomic analysis between the muskrat and 11 other species and identified 6,182 single-copy genes shared by these species (supplementary table S8, Supplementary Material online). A phylogenetic tree was constructed using these genes, with divergence times calculated between each pair of species. We found that the muskrat is sister to a clade of the common ancestor of *Microtus ochrogaster* and *Arvicola amphibius* with a divergence time of 10.8 Ma, which is much later than the divergence time between the muskrat and mouse (fig. 1*C*).

Through a comparison with the common ancestor of the muskrat and mouse, we identified 147 expanded gene families, including 1,191 genes, in the muskrat genome (fig. 1*D*). We performed Gene Ontology (GO) enrichment analysis of these expanded gene families, which showed that they were significantly enriched in 158 GO terms, especially those related to organic synthesis and metabolism, including peptide biosynthetic process (GO:0043043; *P* = 9.24E − 144), peptide metabolic process (GO:0006518; *P* = 9.62E − 126), macromolecule biosynthetic process (GO:0009059; *P* = 1.79E − 96), macromolecule metabolic process (GO:0043170; *P* = 8.55E − 46), organic substance biosynthetic process (GO:1901576; *P* = 1.74E − 73), organic substance metabolic process (GO:0071704; *P* = 5.49E − 30), aromatic compound biosynthetic process (GO:0019438; *P* = 1.47E − 17), heterocycle biosynthetic process (GO:0018130; *P* = 4.50E − 17), organic cyclic compound biosynthetic process (GO:1901362; *P* = 2.10E − 16), and organic cyclic compound metabolic process (GO:1901360; *P* = 1.11E − 17). These significantly enriched GO terms may represent the genomic basis for musk secretion in muskrats. In Kyoto Encyclopedia of Genes and Genomes (KEGG) enrichment analysis, we found 85 significantly enriched pathways, including one reproduction-related pathway, four immune-related pathways, and other pathways related to biological characteristics (supplementary table S9, Supplementary Material online).

## Materials and Methods

### Sample Collection

A male *O. zibethicus* individual was collected from Heilongjiang Harbin Xinke Farm, China for genome assembly. Lung, kidney, muscle, heart, prostate, and scent gland samples were collected from this individual for RNA sequencing. The muscle sample was selected for stLFR sequencing. The liver sample was selected for Hi-C sequencing. We also collected muscle samples from 32 male muskrats from Heilongjiang Harbin Xinke Farm, China for whole-genome resequencing. Sample collection and the related experiments were approved by the Institutional Review Board of BGI (BGI-IRB E21011). All procedures were conducted according to the guidelines of BGI-IRB.

### DNA and RNA Isolation, Library Preparation, and Genome Sequencing

We isolated high-molecular-weight DNA according to the protocol described by Wang et al. (2019), and one stLFR co-barcoding DNA library was constructed using an MGIEasy stLFR Library Prep Kit (MGI, China). The libraries were then sequenced using a BGISEQ-500 sequencer. TRlzol reagent (Invitrogen, USA) was used for total RNA extraction according to the manufacturer's instructions. RNA integrity, purity, and quantity were evaluated using a Qubit 3.0 Fluorometer (Life Technologies, USA) and an Agilent 2100 Bioanalyzer System (Agilent, USA). cDNA libraries were reverse-transcribed using 200–400 bp RNA fragments. Total genomic DNA was extracted using a DNeasy Blood and Tissue Kit (Qiagen, USA). The restriction endonuclease *Mbo*I was used for Hi-C library preparation, and these libraries were subjected to paired-end sequencing via a BGISEQ-500 sequencer (MGI).

### Genome Survey

Jellyfish (v 2.2.6; Marcais and Kingsford 2012) was used to calculate the occurrence of k-mers with short reads prior to genome assembly. In total, 173,452,189,911 k-mers (*K* = 21) were identified, and the peak k-mer depth was 42 (supplementary fig. S1, Supplementary Material online). Results from Jellyfish were inputted into GCE (v1.0.2) to estimate genome size, repeat content, and heterozygosity (Liu et al. 2013).

### Genome Assembly and Assessment

Supernova (v2.1.1; Weisenfeld et al. 2017) was used with its default parameters and stLFR sequencing data to assemble the primary genome. GapCloser (v1.12-r6) and redundans (v0.14a) were used for gap filling and redundancy removal, respectively. Burrows–Wheeler Aligner (BWA, v0.7.17) was used with its with default parameters for mapping Hi-C reads to the initial genome assembly (Li 2013; Pryszcz and Gabaldón 2016; Weisenfeld et al. 2017). Hi-C data quality control was performed via Juicer (v1.5.7; Durand et al. 2016), and 3d-DNA pipeline (v180922) was used to assign contigs at the chromosome level (Durand et al. 2016). The completeness of the gene set and genome were evaluated using BUSCO (v5.2.2; Simão et al. 2015) analysis with the mammalia_odb10 database. Finally, the BGISEQ short reads and Hi-C reads were mapped to our assembled genome using BWA *mem* with its default parameters to calculate the mapping rate.

### Genome Annotation

First, we used long terminal repeat finder (v1.0.6; Xu and Wang 2007), MITE-hunter (v4.07; Han and Wessler 2010), and RepeatModeler2 (v2.0.1; Flynn et al. 2020) to identify de novo repeat motifs. These repeats were then added to the RepBase in RepeatMasker (v4.1.1; Chen, 2004) as known elements for the identification of transposable elements.

Next, we used de novo–, RNA-seq–, and homology-based methods to predict protein-coding genes. The repeat-masked genome was used for de novo gene prediction via GlimmerHMM (v3.0.1), Augustus (v3.0.3), and SNAP (v11/29/2013) (Korf 2004; Majoros et al. 2004; Stanke et al. 2004). The protein sequences of *M. ochrogaster*, *Homo sapiens*, *Rattus norvegicus*, *Mus musculus*, and *Canis lupus familiaris* were used for homology-based gene prediction. The final nonredundant gene set was generated using the MAKER pipeline (v3.01.03) (Campbell et al. 2014) by combining homology, de novo, and RNA-seq supported genes. The completeness of the gene set was evaluated via BUSCO analysis with the mammalia_odb10 database.

### Phylogenetic and Gene Family Analysis

Homologous genes were identified by performing all-to-all BLASTP with proteins from each of the 12 species using the parameter “-evalue 1e-5.” The identified single-copy genes were then used to construct the phylogenetic tree according to the following procedures: (1) multiple amino acid sequence alignments were performed for a single-copy gene orthogroup using MAFFT (Katoh and Standley 2013; v.7.310); (2) PAL2NAL (v14; Suyama et al. 2006) was used to convert the aligned amino acid sequences to DNA sequence alignments; (3) gaps were removed using trimal (v1.4.1; Capella-Gutierrez et al. 2009); (4) a best-fit substitution model was calculated using ModelFinder (Kalyaanamoorthy et al. 2017); and (5) concatenated super genes were used to construct a maximum-likelihood phylogenetic tree via IQTREE (v1.6.12; Nguyen et al. 2015). Gene families were then identified using Treefam (v1.4; Li et al. 2006). Expanded and contracted gene families were detected using CAFÉ (v4.2.1; De Bie et al. 2006). KEGG and GO enrichment analyses were performed on the expanded gene families with all annotated genes used as the background, and Fisher's exact test was used to improve the accuracy of the conducted χ^2^ test. Finally, the Benjamini–Hochberg method (Peng et al. 2017) was used to generate adjusted *P-*values.

### Whole-Genome Sequence Alignment

Whole-genome resequencing data from 32 individuals were mapped to our assembled genome using the BWA *mem* method with its default parameters (Li 2013). The mapping rate and sequencing depth were calculated using SAMtools (v0.1.19; Li et al. 2009) and BamDeal (v0.24; https://github.com/BGI-shenzhen/BamDeal), respectively.

## Supplementary Material

evac138_Supplementary_Data

## Data Availability

The data that support the findings in this study have been deposited into the CNGB Sequence Archive (https://db.cngb.org/cnsa/; Guo et al. 2020) of the China National GeneBank DataBase (Chen et al. 2020) under accession number CNP0003335.

## References

[evac138-B1] Belton J-M , et al 2012. Hi–C: a comprehensive technique to capture the conformation of genomes. Methods 58(3):268–276. 22652625 10.1016/j.ymeth.2012.05.001 PMC3874846

[evac138-B2] Campbell MS , et al 2014. Genome annotation and curation using MAKER and MAKER-P. Curr Protoc Bioinformatics 48(1):4.11. 1–4.11. 39. 25501943 10.1002/0471250953.bi0411s48 PMC4286374

[evac138-B42] Chen N . 2004. Using Repeat Masker to identify repetitive elements in genomic sequences. Curr Protoc Bioinform. 5:4.10. 11–14.10. 14. 10.1002/0471250953.bi0410s05 18428725

[evac138-B3] Cao H , et al 2015. Seasonal expression of prolactin receptor in the scented gland of male muskrat (*Ondatra zibethicus*). Sci Rep. 5:15036. 26477851 10.1038/srep15036 PMC4609948

[evac138-B4] Capella-Gutierrez S , Silla-Martinez JM, Gabaldon T. 2009. Trimal: a tool for automated alignment trimming in large-scale phylogenetic analyses. Bioinformatics 25(15):1972–1973. 19505945 10.1093/bioinformatics/btp348 PMC2712344

[evac138-B5] Chen F , et al 2020. CNGBdb: China National GeneBank DataBase. Hereditas (Beijing) 42:799–809. 10.16288/j.yczz.20-080 32952115

[evac138-B6] De Bie T , et al 2006. CAFE: a computational tool for the study of gene family evolution. Bioinformatics 22(10):1269–1271. 16543274 10.1093/bioinformatics/btl097

[evac138-B7] Durand NC , et al 2016. Juicer provides a one-click system for analyzing loop-resolution Hi-C experiments. Cell Syst. 3(1):95–98. 27467249 10.1016/j.cels.2016.07.002 PMC5846465

[evac138-B8] Flynn JM , et al 2020. Repeatmodeler2 for automated genomic discovery of transposable element families. Proc Natl Acad Sci U S A 117(17):9451–9457. 32300014 10.1073/pnas.1921046117 PMC7196820

[evac138-B9] Guo X , et al 2020. CNSA: a data repository for archiving omics data. Database 2020. 10.1093/database/baaa055 PMC7377928 32705130

[evac138-B10] Han Y , Wessler SR. 2010. MITE-Hunter: a program for discovering miniature inverted-repeat transposable elements from genomic sequences. Nucleic Acids Res. 38(22):e199. 20880995 10.1093/nar/gkq862 PMC3001096

[evac138-B11] Kalyaanamoorthy S , et al 2017. Modelfinder: fast model selection for accurate phylogenetic estimates. Nat Methods 14(6):587–589. 28481363 10.1038/nmeth.4285 PMC5453245

[evac138-B12] Katoh K , Standley DM. 2013. MAFFT Multiple sequence alignment software version 7: improvements in performance and usability. Mol Biol Evol. 30(4):772–780. 23329690 10.1093/molbev/mst010 PMC3603318

[evac138-B13] Kim H , et al 2008. Identification and characterization of potent CYP2B6 inhibitors in Woohwangcheongsimwon suspension, an herbal preparation used in the treatment and prevention of apoplexy in Korea and China. Drug Metab Dispos. 36(6):1010–1015. 18332082 10.1124/dmd.107.019612

[evac138-B14] Korf I . 2004. Gene finding in novel genomes. BMC Bioinf. 5(1):1–9. 10.1186/1471-2105-5-59 PMC421630 15144565

[evac138-B15] Lee D , et al 2019. Neuroprotective effects of musk of muskrat on transient focal cerebral ischemia in rats. Evid Based Complement Alternat Med. 2019:9817949. 31341507 10.1155/2019/9817949 PMC6614976

[evac138-B16] Li B-T , Song F-R. 1994. Determination of chemical composition of muskrat musk. Chin Pharm J. 29:1.

[evac138-B17] Li H , et al 2006. Treefam: a curated database of phylogenetic trees of animal gene families. Nucleic Acids Res. 34(Database issue):D572–D580. 16381935 10.1093/nar/gkj118 PMC1347480

[evac138-B18] Li H , et al 2009. The sequence alignment/map format and SAMtools. Bioinformatics 25(16):2078–2079. 19505943 10.1093/bioinformatics/btp352 PMC2723002

[evac138-B19] Li Y , et al 2017. Comparison of amino acid profiles and metabolic gene expression in muskrat scented glands in secretion and non-secretion season. Sci Rep 7:41158. 28145478 10.1038/srep41158 PMC5286528

[evac138-B20] Li Heng , 2013. Aligning sequence reads, clone sequences and assembly contigs with BWA-MEM. arXiv:1303.3997 [q-bio.GN] 0(0):3.

[evac138-B21] Liu Q , et al 2019. Seasonal expressions of oxytocin and oxytocin receptor in epididymis of the male muskrat (*Ondatra zibethicus*). Theriogenology 124:24–31. 30336300 10.1016/j.theriogenology.2018.10.009

[evac138-B22] Liu B , et al 2013. Estimation of genomic characteristics by analyzing k-mer frequency in de novo genome projects. arXiv preprint arXiv:1308.2012.

[evac138-B23] Majoros WH , Pertea M, Salzberg SL. 2004. Tigrscan and GlimmerHMM: two open source ab initio eukaryotic gene-finders. Bioinformatics 20(16):2878–2879. 15145805 10.1093/bioinformatics/bth315

[evac138-B24] Marcais G , Kingsford C. 2012. Jellyfish: a fast k-mer counter. Tutorialis e Manuais 1:1–8.

[evac138-B25] Nguyen LT , et al 2015. IQ-TREE: a fast and effective stochastic algorithm for estimating maximum-likelihood phylogenies. Mol Biol Evol. 32(1):268–274. 25371430 10.1093/molbev/msu300 PMC4271533

[evac138-B26] Peng J , et al 2017. Multiple confidence intervals for selected parameters adjusted for the false coverage rate in monotone dose-response microarray experiments. Biom J. 59(4):732–745. 28025852 10.1002/bimj.201500254

[evac138-B27] Pizzimenti JJ . 1971. List of karyotypes of mammals from the northern plains region. Trans Kans Acad Sci 74(1):67–75. 5156775

[evac138-B28] Pryszcz LP , Gabaldón T. 2016. Redundans: an assembly pipeline for highly heterozygous genomes. Nucleic Acids Res. 44(12):e113–e113. 27131372 10.1093/nar/gkw294 PMC4937319

[evac138-B29] Schuster RK , Specht P, Rieger S. 2021. On the helminth fauna of the muskrat (*Ondatra zibethicus* (Linnaeus, 1766)) in the Barnim district of Brandenburg state/Germany. Animals (Basel) 11(8):2444. 10.3390/ani11082444. PMC8388652 34438901

[evac138-B30] Simão FA , et al 2015. BUSCO: assessing genome assembly and annotation completeness with single-copy orthologs. Bioinformatics 31(19):3210–3212. 26059717 10.1093/bioinformatics/btv351

[evac138-B31] Skyrienė G , Paulauskas A. 2012. Distribution of invasive muskrats (*Ondatra zibethicus*) and impact on ecosystem. EKOLOGIJA 58:3.

[evac138-B32] Stanke M , et al 2004. AUGUSTUS: a web server for gene finding in eukaryotes. Nucleic Acids Res. 32(suppl_2):W309–W312. 15215400 10.1093/nar/gkh379 PMC441517

[evac138-B33] Suyama M , Torrents D, Bork P. 2006. PAL2NAL: robust conversion of protein sequence alignments into the corresponding codon alignments. Nucleic Acids Res. 34(Web Server issue):W609–W612. 16845082 10.1093/nar/gkl315 PMC1538804

[evac138-B34] van Dorp DA , Klok R, Nugteren DH. 1973. New macrocyclic compounds from the secretions of the civet cat and the musk rat. Recueil des Travaux Chimiques des Pays-Bas 92(8):915–928.

[evac138-B35] Wang O , et al 2019. Efficient and unique cobarcoding of second-generation sequencing reads from long DNA molecules enabling cost-effective and accurate sequencing, haplotyping, and de novo assembly. Genome Res. 29(5):798–808. 30940689 10.1101/gr.245126.118 PMC6499310

[evac138-B36] Ward EM , et al 2021. Muskrats as a bellwether of a drying delta. Commun Biol. 4(1):750. 34168255 10.1038/s42003-021-02288-7 PMC8225612

[evac138-B37] Ward EM , Wysong K, Gorelick SM. 2019. Drying landscape and interannual herbivory-driven habitat degradation control semiaquatic mammal population dynamics. Ecohydrology 13:1.

[evac138-B38] Weisenfeld NI , et al 2017. Direct determination of diploid genome sequences. Genome Res. 27(5):757–767. 28381613 10.1101/gr.214874.116 PMC5411770

[evac138-B39] Xu Z , Wang H. 2007. LTR_FINDER: an efficient tool for the prediction of full-length LTR retrotransposons. Nucleic Acids Res 35(Web Server issue):W265–W268. 17485477 10.1093/nar/gkm286 PMC1933203

[evac138-B40] Zhang L , Zhang H, Hua Y. 2020. Evolutionary status of the invasive muskrat *Ondatra zibethicus* revealed by complete mitochondrial genome. Mitochondrial DNA B Resour. 5(1):980–981. 33366836 10.1080/23802359.2020.1719931 PMC7748713

[evac138-B41] Zhou C , et al 2020. Genomic evidence sheds light on the genetic mechanisms of musk secretion in muskrats. Int J Biol Macromol. 145:1189–1198. 31726118 10.1016/j.ijbiomac.2019.10.045

