# Supplementary material for: Chromosome-level Genome of the Muskrat (Ondatra zibethicus)
Source: Genome Biol Evol. 2022 Sep 16;14(10):evac138. doi: 10.1093/gbe/evac138 (PMC9539402; doi:10.1093/gbe/evac138)
Supplement: evac138_Supplementary_Data [file evac138_supplementary_data.docx]

**Table S1** Comparison of assembly statistics among our assembled *O. zibethicus* genome, the previously published *O. zibethicus* genome.

| Assembly Level | Parameters | Our assembled genome | OndZib_v1_BIUU |
| --- | --- | --- | --- |
| Scaffold | Maximal length (bp) | 196,464,644 | 1,045,473 |
|  | N90 (bp) | 52,000 | 9124 |
|  | N50 (bp) | 80,254,335 | 89,093 |
|  | number>=100bp | 63,351 | 565,356 |
|  | number>=2kb | 36,058 | 54,985 |
|  | Ratio of Ns | 0.095 | 0.067 |
|  | Genome size (bp) | 2,631,401,031 | 2,562,752,769 |
| Contig | Maximal length (bp) | 704,436 | 743,410 |
|  | N90 (bp) | 11,543 | 8504 |
|  | N50 (bp) | 66,830 | 73,746 |
|  | number>=100bp | 116,040 | 571,599 |
|  | number>=2kb | 78,215 | 61,228 |
|  | Genome size (bp) | 2,382,627,411 | 2,562,128,469 |

**Table S2** Result of BUSCO analysis of the *O. zibethicus* genome.

| Level | BUSCO assessment results | Dataset | Parameter |
| --- | --- | --- | --- |
| Genome | C:91.3% [S:89.5%, D:1.8%], F:3.3%, M:5.4%, n:9226 | mammalia_odb10 | -m genome |
| Gene | C:91.3% [S:90.0%, D:1.3%], F:3.5%, M:5.2%, n:9226 | mammalia_odb10 | -m proteins |

**Table S3** Statistics of Repeats in the *O. zibethicus* genome.

| **Type** | **Length (bp)** | **% of genome** |
| --- | --- | --- |
| Trf | 54,476,133 | 2.07 |
| Repeatmasker | 578,314,576 | 21.98 |
| Proteinmask | 147,706,609 | 5.61 |
| Denovo | 669,905,498 | 25.46 |
| Total | 904,506,239 | 34.37 |

**Table S4** Statistics of identified Repeats by *De novo* method in *O. zibethicus* Genome.

| **Type** | **Length (bp)** | **% of genome** |
| --- | --- | --- |
| DNA | 3,875,293 | 0.15 |
| LINE | 450,220,459 | 17.11 |
| SINE | 126,243,211 | 4.80 |
| LTR | 169,681,460 | 6.45 |
| Other | 900,582 | 0.03 |
| Satellite | 187,813 | 0.01 |
| Simple_repeat | 1,298,169 | 0.05 |
| Unknown | 1,106,442 | 0.04 |
| Total | 669,905,498 | 25.46 |

**Table S5** Transposable elements in the *O. zibethicus* genome assembly.

|  | **Repbase TEs** | | **TE proteins** | | ***De novo*** | | **Combined TEs** | |
| --- | --- | --- | --- | --- | --- | --- | --- | --- |
| **Type** | Length (bp) | % in genome | Length (bp) | % in genome | Length (bp) | % in genome | Length (bp) | % in genome |
| **DNA** | 28,299,052 | 1.08 | 158,677 | 0.01 | 3,875,293 | 0.15 | 31,109,354 | 1.18 |
| **LINE** | 268,909,493 | 10.22 | 120,647,652 | 4.58 | 450,220,459 | 17.11 | 567,283,056 | 21.56 |
| **SINE** | 113,796,803 | 4.32 | 0 | 0 | 126,243,211 | 4.80 | 191,635,156 | 7.28 |
| **LTR** | 169,834,272 | 6.45 | 26,936,698 | 1.02 | 169,681,460 | 6.45 | 286,487,486 | 10.89 |
| **Other** | 7,192,676 | 0.27 | 171 | 0.00 | 900,582 | 0.03 | 7,232,856 | 0.27 |
| **Unknown** | 0 | 0 | 0 | 0 | 1,106,442 | 0.04 | 1,106,442 | 0.04 |
| **Total** | 578,314,576 | 21.98 | 147,706,609 | 5.61 | 668,419,516 | 25.40 | 876,685,433 | 33.32 |

Note: Repbase TEs: the result of RepeatMasker based on Repbase; TE proteins: the result of RepeatProteinMask based on Repbase; *De novo*: *de novo* finding repeats (Repeatmodeler and LTR_FINDING); Combined TEs: the results obtained from combining the results using all the approaches.

**Table S6** Statistics on functional annotation of the *O. zibethicus* gene set.

| **Values** | **Total** | **Swissprot-Annotated** | **KEGG-Annotated** | **TrEMBL-Annotated** | **Interpro-Annotated** | **GO-Annotated** | **Overall** |
| --- | --- | --- | --- | --- | --- | --- | --- |
| **Number** | 19,396 | 19,268 | 17,613 | 19,372 | 19,339 | 14,334 | 19,395 |
| **Percentage** | 100% | 99.34% | 90.81% | 99.88% | 99.71% | 73.90% | 99.99% |

**Table S7** Statistics of ncRNA annotation.

| **Type** |  | **number** | **Average length (bp)** | **Total length (bp)** | **% of genome** |
| --- | --- | --- | --- | --- | --- |
| **miRNA** | | 775 | 96.99483871 | 75,171 | 0.002857 |
| **tRNA** | | 3685 | 72.96933514 | 268,892 | 0.010219 |
| **rRNA** | rRNA | 618 | 88.92718447 | 54,957 | 0.002089 |
|  | 18S | 32 | 183.4375 | 5870 | 0.000223 |
|  | 28S | 110 | 116.6181818 | 12,828 | 0.000487 |
|  | 5.8S | 2 | 145.5 | 291 | 0.000011 |
|  | 5S | 474 | 75.88185654 | 35,968 | 0.001367 |
| **snRNA** | snRNA | 1559 | 118.958948 | 185,457 | 0.007048 |
|  | CD-box | 194 | 91.5257732 | 17,756 | 0.000675 |
|  | HACA-box | 215 | 136.0372093 | 29,248 | 0.001111 |
|  | splicing | 1104 | 119.5271739 | 131,958 | 0.005015 |

**Table S8** Species used in comparative genomics analysis.

| **Species** | **Short Name** | **Database** | **Version** |
| --- | --- | --- | --- |
| *Arvicola amphibius* | *A. amphibius* | NCBI | mArvAmp1.2 |
| *Bos taurus* | *B. taurus* | Ensembl | ARS-UCD1.2 |
| *Canis lupus familiaris* | *C. lupus familiaris* | Ensembl | CanFam3.1 |
| *Castor canadensis* | *C. canadensis* | Ensembl | C.can_genome_v1.0 |
| *Felis catus* | *F. catus* | Ensembl | Felis_catus_9.0 |
| *Homo sapiens* | *H. sapiens* | Ensembl | GRCh38 |
| *Microtus ochrogaster* | *M. ochrogaster* | NCBI | MicOch1.0 |
| *Monodelphis domestica* | *M. domestica* | Ensembl | ASM229v1 |
| *Moschus moschiferus* | *M. moschiferus* | Ensembl | MosMos_v2_BIUU_UCD |
| *Mus musculus* | *M. musculus* | Ensembl | GRCm38 |
| *Octodon degus* | *O. degus* | Ensembl | OctDeg1.0 |
| *Ondatra zibethicus* | *O. zibethicus* | *--* | this study |

**Table S9** The results of the GO enrichment analysis of expanded gene families in the muskrat genome.

| ontology | ID | GO Term | Adjusted P value |
| --- | --- | --- | --- |
| cellular component | GO:0005840 | ribosome | 3.30E-174 |
| molecular function | GO:0003735 | structural constituent of ribosome | 7.23E-174 |
| biological process | GO:0006412 | translation | 2.42E-145 |
| biological process | GO:0043043 | peptide biosynthetic process | 9.24E-144 |
| biological process | GO:0043604 | amide biosynthetic process | 1.62E-138 |
| molecular function | GO:0005198 | structural molecule activity | 2.16E-137 |
| cellular component | GO:1990904 | ribonucleoprotein complex | 5.41E-136 |
| biological process | GO:0006518 | peptide metabolic process | 9.62E-126 |
| biological process | GO:0043603 | cellular amide metabolic process | 1.35E-111 |
| biological process | GO:0034645 | cellular macromolecule biosynthetic process | 8.44E-97 |
| biological process | GO:0009059 | macromolecule biosynthetic process | 1.79E-96 |
| biological process | GO:0044271 | cellular nitrogen compound biosynthetic process | 3.13E-95 |
| biological process | GO:0034641 | cellular nitrogen compound metabolic process | 9.80E-86 |
| biological process | GO:0015074 | DNA integration | 3.93E-78 |
| biological process | GO:0044249 | cellular biosynthetic process | 5.83E-76 |
| biological process | GO:1901566 | organonitrogen compound biosynthetic process | 3.49E-75 |
| molecular function | GO:0004518 | nuclease activity | 3.15E-74 |
| biological process | GO:1901576 | organic substance biosynthetic process | 1.74E-73 |
| biological process | GO:0006259 | DNA metabolic process | 4.36E-73 |
| biological process | GO:0009058 | biosynthetic process | 4.55E-71 |
| cellular component | GO:0043228 | non-membrane-bounded organelle | 2.27E-67 |
| cellular component | GO:0043232 | intracellular non-membrane-bounded organelle | 2.27E-67 |
| molecular function | GO:0004523 | RNA-DNA hybrid ribonuclease activity | 7.82E-62 |
| biological process | GO:0051704 | multi-organism process | 3.37E-59 |
| biological process | GO:0044260 | cellular macromolecule metabolic process | 5.30E-57 |
| molecular function | GO:0016891 | endoribonuclease activity, producing 5'-phosphomonoesters | 9.31E-57 |
| molecular function | GO:0016893 | endonuclease activity, active with either ribo- or deoxyribonucleic acids and producing 5'-phosphomonoesters | 3.66E-55 |
| biological process | GO:0016032 | viral process | 3.65E-53 |
| biological process | GO:0044403 | symbiont process | 3.65E-53 |
| biological process | GO:0044419 | interspecies interaction between organisms | 3.65E-53 |
| molecular function | GO:0004521 | endoribonuclease activity | 8.37E-52 |
| molecular function | GO:0016779 | nucleotidyltransferase activity | 1.99E-50 |
| biological process | GO:0010467 | gene expression | 2.82E-48 |
| cellular component | GO:0044444 | cytoplasmic part | 6.17E-48 |
| biological process | GO:0043170 | macromolecule metabolic process | 8.55E-46 |
| molecular function | GO:0003964 | RNA-directed DNA polymerase activity | 1.24E-45 |
| biological process | GO:0006278 | RNA-dependent DNA biosynthetic process | 2.15E-44 |
| molecular function | GO:0004540 | ribonuclease activity | 1.02E-43 |
| molecular function | GO:0004519 | endonuclease activity | 2.38E-43 |
| cellular component | GO:0005737 | cytoplasm | 4.69E-39 |
| biological process | GO:0006807 | nitrogen compound metabolic process | 2.36E-38 |
| molecular function | GO:0140097 | catalytic activity, acting on DNA | 5.66E-38 |
| biological process | GO:0071897 | DNA biosynthetic process | 6.32E-38 |
| molecular function | GO:0034061 | DNA polymerase activity | 3.53E-35 |
| biological process | GO:0044238 | primary metabolic process | 2.05E-34 |
| biological process | GO:0044237 | cellular metabolic process | 6.27E-33 |
| molecular function | GO:0140098 | catalytic activity, acting on RNA | 3.31E-32 |
| biological process | GO:0071704 | organic substance metabolic process | 5.49E-30 |
| molecular function | GO:0003676 | nucleic acid binding | 1.64E-29 |
| biological process | GO:0008152 | metabolic process | 4.23E-29 |
| molecular function | GO:0004190 | aspartic-type endopeptidase activity | 8.32E-27 |
| molecular function | GO:0070001 | aspartic-type peptidase activity | 8.32E-27 |
| cellular component | GO:0032991 | protein-containing complex | 4.66E-25 |
| biological process | GO:0090304 | nucleic acid metabolic process | 2.99E-23 |
| biological process | GO:0006139 | nucleobase-containing compound metabolic process | 3.25E-20 |
| biological process | GO:0006725 | cellular aromatic compound metabolic process | 9.29E-19 |
| biological process | GO:0046483 | heterocycle metabolic process | 1.03E-18 |
| biological process | GO:0034654 | nucleobase-containing compound biosynthetic process | 2.46E-18 |
| molecular function | GO:0008270 | zinc ion binding | 4.63E-18 |
| biological process | GO:1901360 | organic cyclic compound metabolic process | 1.11E-17 |
| biological process | GO:0019438 | aromatic compound biosynthetic process | 1.47E-17 |
| biological process | GO:0018130 | heterocycle biosynthetic process | 4.50E-17 |
| biological process | GO:1901362 | organic cyclic compound biosynthetic process | 2.10E-16 |
| biological process | GO:0019538 | protein metabolic process | 4.48E-16 |
| biological process | GO:0044267 | cellular protein metabolic process | 1.96E-14 |
| cellular component | GO:0043229 | intracellular organelle | 7.78E-14 |
| cellular component | GO:0043226 | organelle | 2.98E-13 |
| molecular function | GO:0046914 | transition metal ion binding | 1.15E-10 |
| biological process | GO:0006414 | translational elongation | 1.88E-10 |
| biological process | GO:1901564 | organonitrogen compound metabolic process | 4.44E-10 |
| cellular component | GO:0015934 | large ribosomal subunit | 1.12E-09 |
| cellular component | GO:0005622 | intracellular | 1.60E-09 |
| cellular component | GO:0044424 | intracellular part | 1.60E-09 |
| molecular function | GO:0004459 | L-lactate dehydrogenase activity | 2.03E-09 |
| molecular function | GO:0004457 | lactate dehydrogenase activity | 2.03E-09 |
| molecular function | GO:0016788 | hydrolase activity, acting on ester bonds | 3.00E-09 |
| cellular component | GO:0044391 | ribosomal subunit | 3.39E-09 |
| biological process | GO:0006355 | regulation of transcription, DNA-templated | 5.56E-09 |
| biological process | GO:1903506 | regulation of nucleic acid-templated transcription | 5.56E-09 |
| biological process | GO:2001141 | regulation of RNA biosynthetic process | 5.56E-09 |
| biological process | GO:0051252 | regulation of RNA metabolic process | 2.29E-08 |
| molecular function | GO:0003746 | translation elongation factor activity | 2.90E-08 |
| biological process | GO:2000112 | regulation of cellular macromolecule biosynthetic process | 3.56E-08 |
| biological process | GO:0010556 | regulation of macromolecule biosynthetic process | 3.73E-08 |
| biological process | GO:0031326 | regulation of cellular biosynthetic process | 4.54E-08 |
| biological process | GO:0009889 | regulation of biosynthetic process | 4.70E-08 |
| biological process | GO:0019219 | regulation of nucleobase-containing compound metabolic process | 4.70E-08 |
| biological process | GO:0019068 | virion assembly | 7.58E-08 |
| cellular component | GO:0030286 | dynein complex | 3.98E-07 |
| biological process | GO:0031323 | regulation of cellular metabolic process | 4.10E-07 |
| biological process | GO:0019058 | viral life cycle | 6.31E-07 |
| molecular function | GO:0004807 | triose-phosphate isomerase activity | 7.73E-07 |
| biological process | GO:0006351 | transcription, DNA-templated | 1.32E-06 |
| biological process | GO:0097659 | nucleic acid-templated transcription | 1.32E-06 |
| biological process | GO:0010468 | regulation of gene expression | 1.50E-06 |
| biological process | GO:0032774 | RNA biosynthetic process | 1.55E-06 |
| biological process | GO:0051171 | regulation of nitrogen compound metabolic process | 5.64E-06 |
| biological process | GO:0019222 | regulation of metabolic process | 6.96E-06 |
| biological process | GO:0080090 | regulation of primary metabolic process | 7.09E-06 |
| molecular function | GO:0008569 | ATP-dependent microtubule motor activity, minus-end-directed | 1.19E-05 |
| cellular component | GO:0005623 | cell | 1.19E-05 |
| cellular component | GO:0044464 | cell part | 1.19E-05 |
| molecular function | GO:0008097 | 5S rRNA binding | 1.53E-05 |
| cellular component | GO:0005875 | microtubule associated complex | 1.84E-05 |
| biological process | GO:0060255 | regulation of macromolecule metabolic process | 4.99E-05 |
| molecular function | GO:0045505 | dynein intermediate chain binding | 8.59E-05 |
| molecular function | GO:0051959 | dynein light intermediate chain binding | 8.59E-05 |
| molecular function | GO:0031492 | nucleosomal DNA binding | 0.000133 |
| molecular function | GO:0031490 | chromatin DNA binding | 0.000133 |
| molecular function | GO:0016861 | intramolecular oxidoreductase activity, interconverting aldoses and ketoses | 0.000133 |
| molecular function | GO:0004175 | endopeptidase activity | 0.000299 |
| biological process | GO:0009987 | cellular process | 0.00032 |
| molecular function | GO:0031491 | nucleosome binding | 0.000365 |
| biological process | GO:0002182 | cytoplasmic translational elongation | 0.000421 |
| biological process | GO:0008150 | biological process | 0.000608 |
| molecular function | GO:1901363 | heterocyclic compound binding | 0.000646 |
| molecular function | GO:0097159 | organic cyclic compound binding | 0.00073 |
| molecular function | GO:0001730 | 2'-5'-oligoadenylate synthetase activity | 0.000781 |
| biological process | GO:0002181 | cytoplasmic translation | 0.000781 |
| molecular function | GO:0051537 | 2 iron, 2 sulfur cluster binding | 0.000809 |
| cellular component | GO:0005758 | mitochondrial intermembrane space | 0.001592 |
| cellular component | GO:0031970 | organelle envelope lumen | 0.001592 |
| molecular function | GO:0005248 | voltage-gated sodium channel activity | 0.002074 |
| biological process | GO:0010506 | regulation of autophagy | 0.002822 |
| molecular function | GO:0008135 | translation factor activity, RNA binding | 0.004336 |
| molecular function | GO:0004726 | non-membrane spanning protein tyrosine phosphatase activity | 0.00585 |
| cellular component | GO:0001518 | voltage-gated sodium channel complex | 0.00593 |
| cellular component | GO:0034706 | sodium channel complex | 0.00593 |
| molecular function | GO:0005219 | ryanodine-sensitive calcium-release channel activity | 0.007766 |
| molecular function | GO:0016860 | intramolecular oxidoreductase activity | 0.008493 |
| molecular function | GO:0003824 | catalytic activity | 0.013773 |
| molecular function | GO:1990939 | ATP-dependent microtubule motor activity | 0.015148 |
| molecular function | GO:0003725 | double-stranded RNA binding | 0.016072 |
| cellular component | GO:0016272 | prefoldin complex | 0.016528 |
| molecular function | GO:0003777 | microtubule motor activity | 0.017635 |
| molecular function | GO:0019843 | rRNA binding | 0.01922 |
| cellular component | GO:0015630 | microtubule cytoskeleton | 0.020703 |
| cellular component | GO:0022625 | cytosolic large ribosomal subunit | 0.022875 |
| molecular function | GO:0005272 | sodium channel activity | 0.022875 |
| cellular component | GO:0022626 | cytosolic ribosome | 0.022875 |
| cellular component | GO:0044445 | cytosolic part | 0.026191 |
| biological process | GO:0061817 | endoplasmic reticulum-plasma membrane tethering | 0.027827 |
| molecular function | GO:0003993 | acid phosphatase activity | 0.027827 |
| cellular component | GO:0005854 | nascent polypeptide-associated complex | 0.027827 |
| biological process | GO:0051643 | endoplasmic reticulum localization | 0.027827 |
| biological process | GO:0051607 | defense response to virus | 0.03133 |
| biological process | GO:0006749 | glutathione metabolic process | 0.03133 |
| molecular function | GO:0015278 | calcium-release channel activity | 0.034473 |
| molecular function | GO:0099604 | ligand-gated calcium channel activity | 0.034473 |
| molecular function | GO:0003682 | chromatin binding | 0.034886 |
| biological process | GO:0031329 | regulation of cellular catabolic process | 0.034886 |
| biological process | GO:0009615 | response to virus | 0.03583 |
| molecular function | GO:0005201 | extracellular matrix structural constituent | 0.041039 |
| molecular function | GO:0070566 | adenylyltransferase activity | 0.041039 |
| molecular function | GO:0042043 | neurexin family protein binding | 0.041145 |
| molecular function | GO:0004749 | ribose phosphate diphosphokinase activity | 0.041145 |
| cellular component | GO:0031083 | BLOC-1 complex | 0.041145 |
| cellular component | GO:0031082 | BLOC complex | 0.041145 |


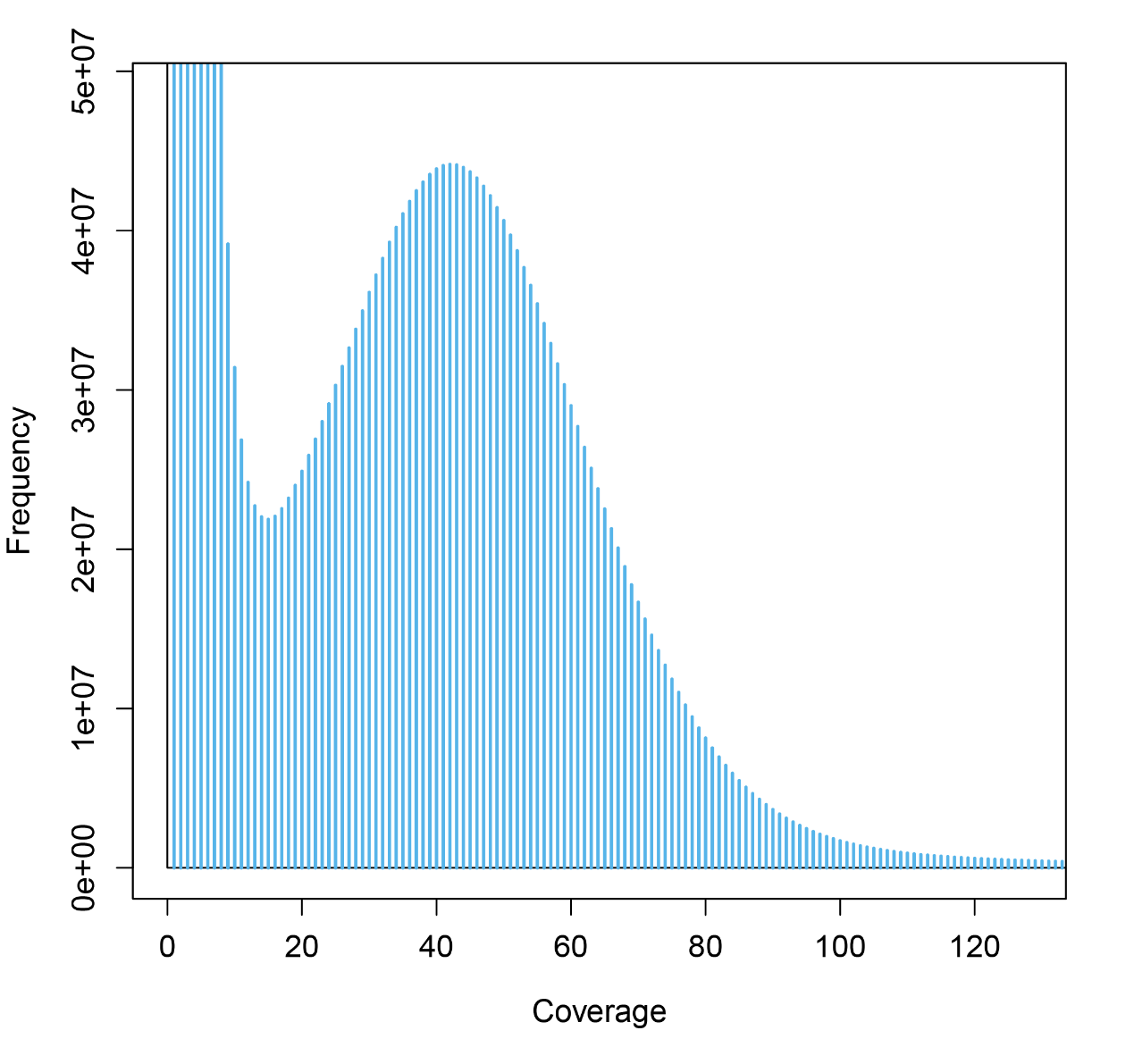


**Fig S1.** K-mer frequency distribution at k-mer size of 21. K-mer refers to an artificial sequence division of K nucleotides.


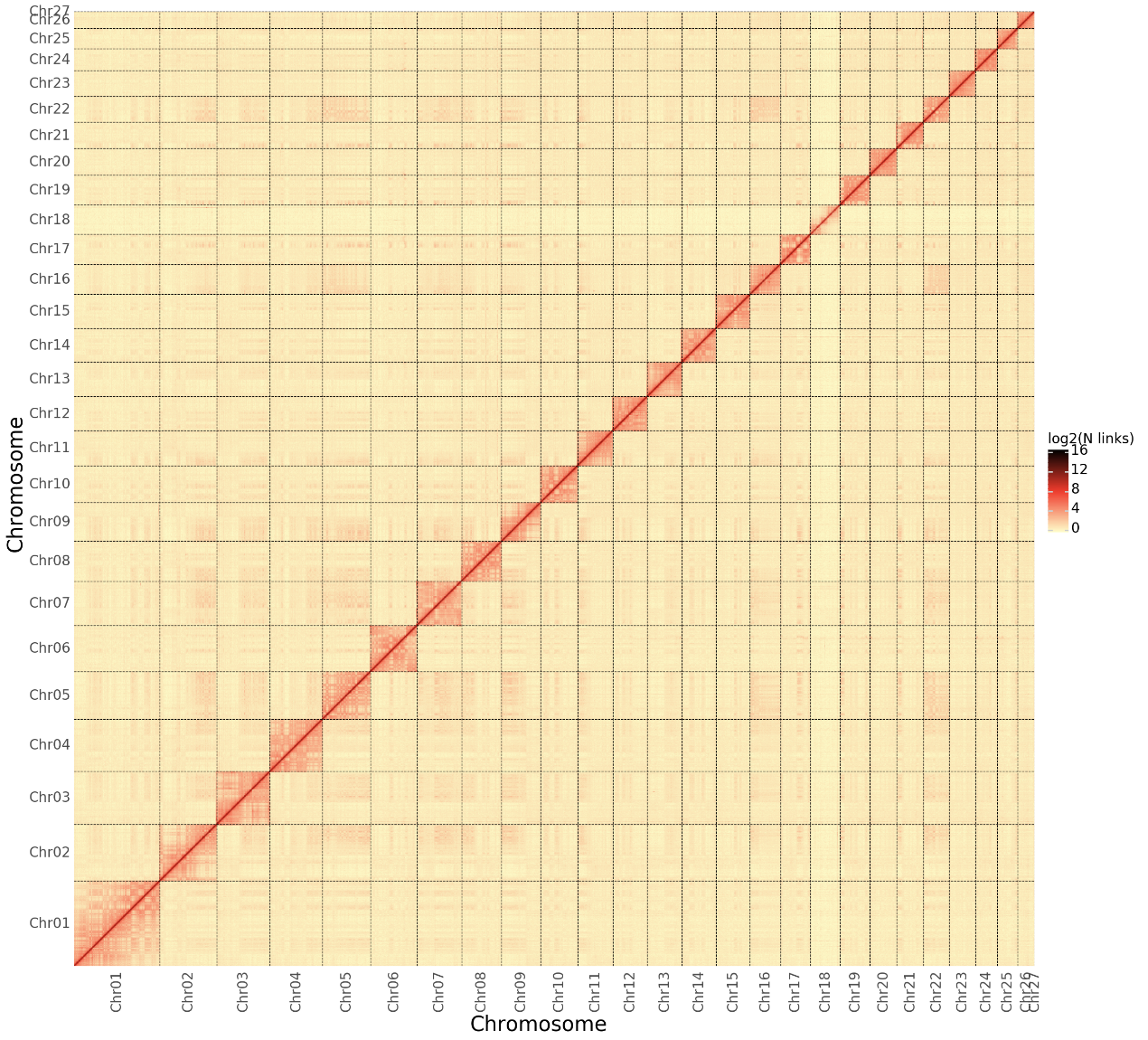


**Fig S2.** Scaffold contact matrix of the *O. zibethicus* genome in this study. The density of Hi-C interactions was represented by the color depth.


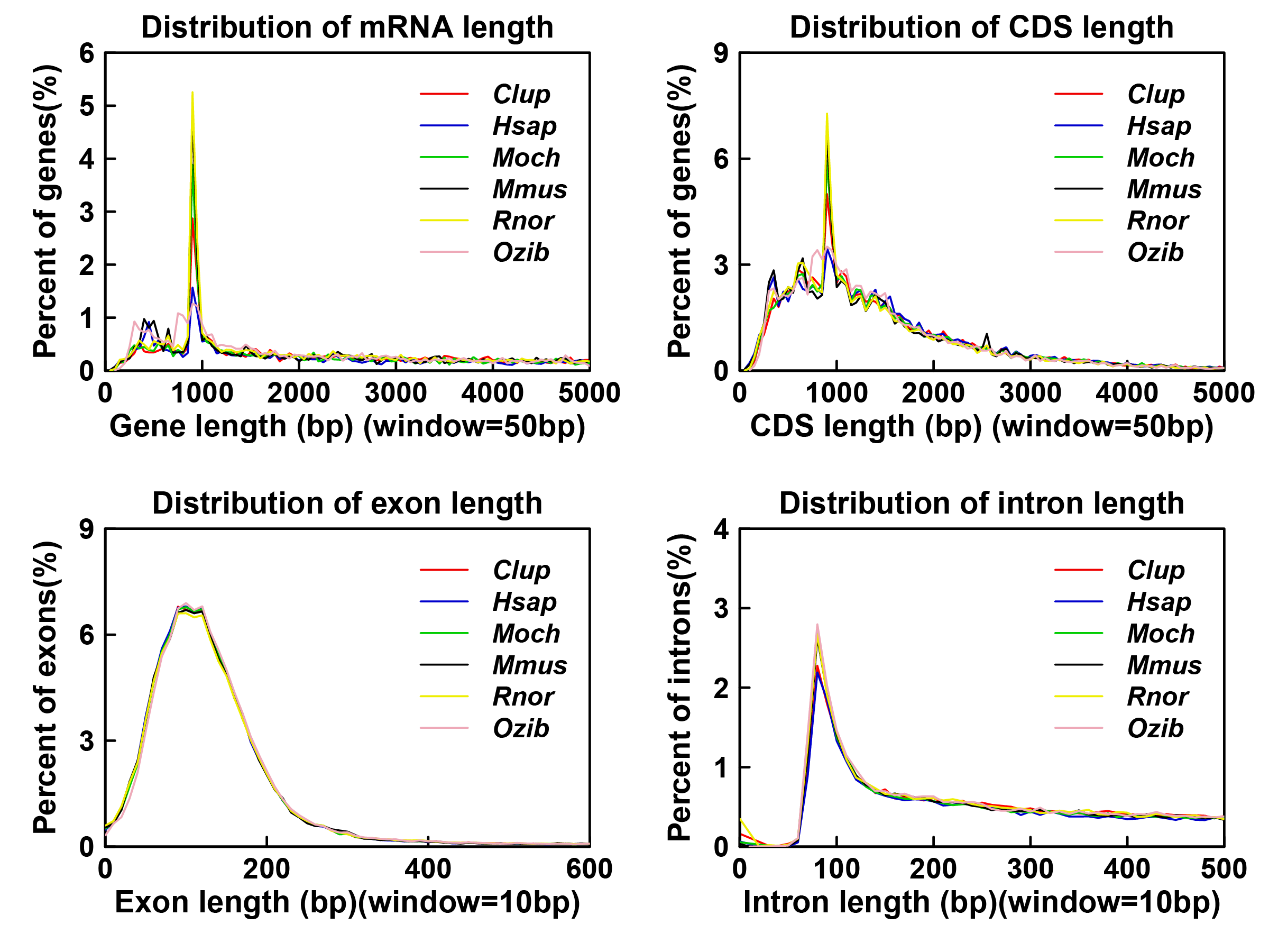


**Fig S3.** Comparisons of CDS length, mRNA length, intron length and exon length among 6 species. Clup: dog; Hsap: human; Moch: prairie vole; Mmus: mouse; Rnor: rat; Ozib: muskrat;


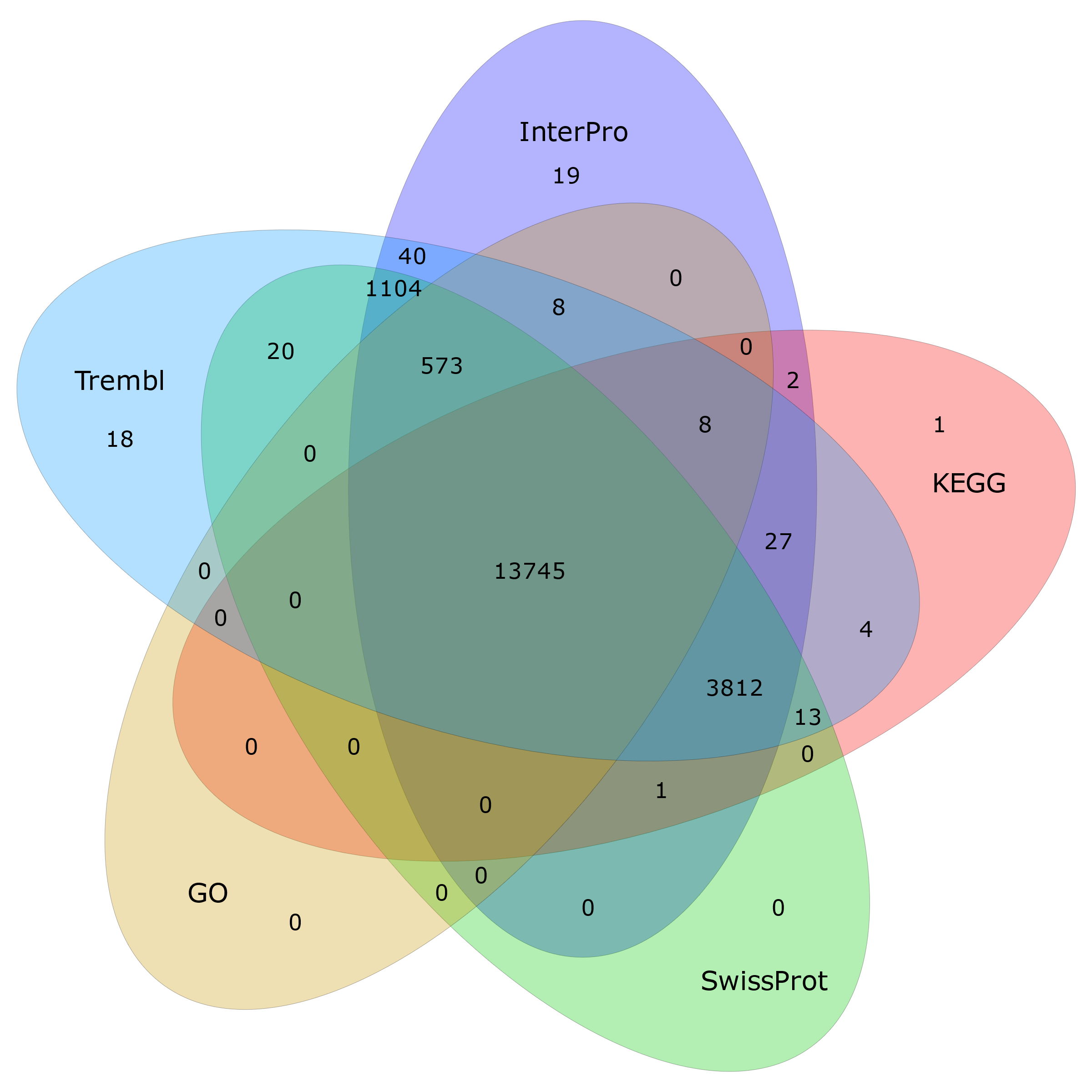


**Fig S4.** Venn diagram representing the functional annotation of the *O. zibethicus* gene set.
